# Geochemical composition and potential health risks of geophagic materials: an example from a rural area in the Limpopo Province of South Africa

**DOI:** 10.1007/s10653-023-01551-6

**Published:** 2023-06-09

**Authors:** Hassina Mouri, Retshepile Evelyn Malepe, Carla Candeias

**Affiliations:** 1 Department of Geology, University of Johannesburg, Johannesburg, South Africa; 2 GeoBioTec Research Unit, Geosciences Department, University of Aveiro, 3810-193 Aveiro, Portugal

**Keywords:** Geophagic materials, Bioaccessible fraction, Health risk assessment, South Africa

## Abstract

Geophagy is a common practice among rural population of the Fetakgomo Tubatse Local Municipality area in the Limpopo Province of South Africa. Although, the practice might be beneficial to the health of the consumers, its negative effects could overshadow the positive effects and might lead to detrimental health issues. The present work sought to investigate the geochemical composition as well as pH and organic matter (OM) content of geophagic materials commonly consumed in the study area. Furthermore, assessment of the potential health risk of the materials on geophagic individuals was also considered. Twelve samples were collected in the study area and analysed by X-ray Fluorescence (XRF) and Inductively Coupled Plasma—Mass Spectrometry (ICP-MS) for major and trace elements composition. The results showed higher concentrations of non-essential elements (e.g., As, Cr, Pb) than the proposed recommended daily standards intake, suggesting a potential health risk. The alkaline nature (pH 6.80 to 9.22) of the studied samples might affect the bioacessibility of some essential elements. Furthermore, the OM content (> 0.7%) observed in some of the studied samples may retain pathogenic micro-organisms detrimental to health. Although As and Cr presented a low bioaccessible fraction (< 16.0%), health risk assessment revealed that their concentrations represented a hazard (HQ > 1) and might induce non-carcinogenic health threats to geophagic individuals. Based on the geochemical analysis, pH and OM content as well as health risk assessment findings, the studied geophagic materials are not considered suitable for human consumption. The practice should therefore be discouraged amongst the population in the study area to avoid possible detrimental health issues.

**Supplementary Information:**

The online version contains supplementary material available at 10.1007/s10653-023-01551-6.

## Introduction

Geophagy is commonly described as the deliberate practice of consuming earthy materials such as rocks, soils, and clays by humans and animals (Ekosse et al., 2017). The practice cuts across individuals of different age groups, gender, ethnicity, and socio-economic classes mainly associated with pregnancy (Kambunga et al., 2019a, 2019b). Recent studies in Limpopo Province, South Africa, revealed that geophagy is equally reported among adolescents, non-pregnant women, and men (Mashao et al., 2021; Phakoago et al., 2019). Geophagy is widespread where its prevalence is entrenched in African countries, such as South Africa, Namibia, Nigeria, Ghana, and Kenya (De Jager et al., 2013; Gevera & Mouri, 2021; Kambunga et al., 2019a, 2019b; Momoh et al., 2015).

Geophagic materials are commonly consumed based on several reasons, including nutrients supplementation to compensate the deficiency of some essential elements such as Fe, Ca, Mg, Zn in the human diet (Kambunga et al., 2019a, 2019b; Lakudzala & Khonje, 2011); medicinal properties used as remedy to cure some common illnesses in the gastrointestinal (GI) tract (e.g., diarrhoea and constipation) (Ekosse et al., 2017; Fosso-Kankeu et al., 2015); and to ease cravings and morning sicknesses as part of cultural and traditional beliefs among pregnant women (Kambunga et al., 2019a, 2019b; Msibi, 2014).

Despite beliefs in the benefits of geophagy, the practice presents detrimental health risk to geophagic individuals. Studies by Ekosse and Anyangwe (2012) revealed that geophagic materials have the potential to decrease the body absorption of elements. The latter could lead to micronutrients deficiencies such as Fe deficiency (Mogongoa et al., 2011). The presence of potentially toxic elements (PTEs), such as As, Co, and Pb, in the consumed materials could pose both carcinogenic and non-carcinogenic health risks. Therefore, the practice of geophagy can be rather harmful to the health of geophagic individuals in a longer term, depending on the nature and composition of the consumed materials (Gevera & Mouri, 2021).

The prevalence of geophagy in the rural area of the Fetakgomo Tubatse Local Municipality (FTLM) in Limpopo Province of South Africa is widespread. A substantial amount of geophagic materials can be consumed daily by the residents in the area regardless of their gender. However, due to cultural reasons, only women declared openly their practice during a geophagy interview questionnaires (Malepe, 2022). Like in many other countries in Africa (Kambunga et al., 2019a, 2019b) and in South Africa (Malepe et al., 2023), geophagy among women in the study area, is motivated by several factors, which are both sociocultural and physiological. These factors include cultural and traditional beliefs, need for supplementation of nutrients deficiency especially during pregnancy, detoxification, and protection of the gastrointestinal tract from toxins and overacidity (Malepe et al., 2023).

The choice of the consumed material in the study area is controlled mostly by the availability of the material locally, easy accessibility in the environment at no cost and no difficulties to acquire. However, other factors that can control the choice and attraction to the type of the consumed material include taste, colour, smell and texture for example, which can also vary from one individual to another based on the needs for consumption (Malepe, 2022).

Despite the widespread of geophagy in the study area, no studies have been undertaken to assess the composition of the consumed materials as well as their potential health effects. The present study sought to assess the geochemical composition of geophagic materials commonly consumed in the FTLM area, as well as their pH, organic matter (OM) content, non-carcinogenic health risk, and bioaccessible fraction, and infer their associated potential health risk to geophagic individuals.

## Materials and methods

### Study area and geology

**Fig. 1:**
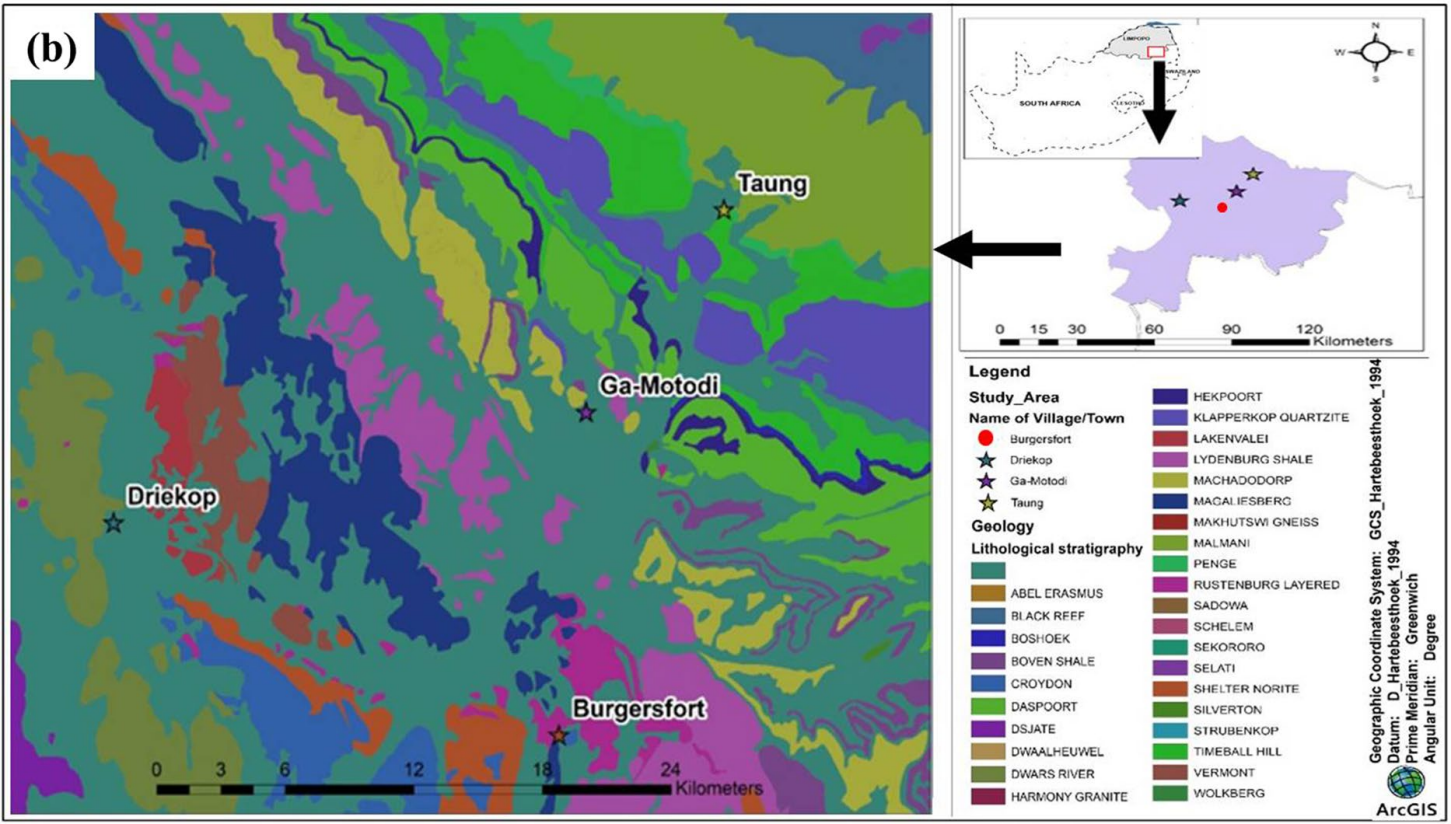
Geological map of the Fetakgomo Tubatse Local Municipality area showing the locations of the collected samples (Driekop, Ga-Motodi, and Taung) in Limpopo Province, South Africa. (adapt. Council for Geoscience, shapefiles 1:1000 000 Geological Map, 2018)

The Fetakgomo Tubatse Local Municipality (FTLM) lies in the Sekhukhune District Municipality of the Limpopo Province of South Africa. It is bounded between the N4 highway of Middelburg in the Mpumalanga Province in the north and N1 highway of Polokwane in Limpopo Province in the east. Driekop (S 24°35′42.00", E 30°8′52.80"), Ga-Motodi (S 24°33′0.84", E 30°20′47.22") and Taung (S 24°28′4.30", E 30°24′14.66") are the three localities within the Burgersfort town, where the studied geophagic materials were collected (Fig. 1).

From a geological point of view, the studied samples were collected in an area that is dominated by rocks of the Transvaal Basin representing a succession of chemical (Chuniespoort Group) and clastic sedimentary rocks with minor volcanic rocks (Pretoria Group) (Eriksson et al., 2006). The Chuniespoort Group comprises mainly of carbonate rocks and banded iron formations (BIFs) (Warke, 2017), whereas the Pretoria Group represents the three weathering resistant quartzite formations (i.e., Magaliesberg, Daspoortrand and Timeball Hill) underlain by shales and lavas in between (Bumby et al., 2012; Eriksson et al., 2001). A layered sequence of mafic–ultramafic rocks in eastern limb of the Rustenburg Layered Suite (RLS) with rock units such as diorite, gabbro, magnetite, chromitite and anorthosite (Cawthorn et al., 2006) also form part of the formation within the study localities. Owing to its geological nature, minerals (such as andalusite and asbestos) and chemical elements (such as magnetite, chrome, platinum, silica, and vanadium) are found in abundance resulting in many establishments of mining operations in the area (FTLM Integrated Development Plan, 2019/2020).

### Description of the study material

Studied geophagic materials are commonly collected from the yard homes, hills, mining dumps and riverbeds without monetary cost, easily accessible and readily available for consumption by the communities around. A total of 12 representative geophagic samples weighing between 2 to 3 kg were randomly collected close to the surface at the same sites and depth as where they are commonly collected by geophagic individuals. The samples considered for this study were collected from 3 different localities called Driekop, Ga-Motodi and Taung (Fig. 1).

- (i) Driekop locality – 4 samples (DRI-01 to DRI-04) were collected from this locality. Except for sample DRI-01 collected from the yard characterized by medium texture and white color (Fig. S1a), other samples DRI-02 to DRI-04 were collected from the mining dumps characterized by coarse-grained texture and dark brownish color (Fig. S1b).
- (ii) Ga-Motodi locality – 5 samples (GAM-01 to GAM-05) were collected at the riverbeds except for sample GAM-04, it was collected from the yard. All samples from this locality, showed similar fine-grained texture, with colors varying from grey (GAM-01; Fig. S2a), dark brown (GAM-02; Fig. S2b), dark reddish-brown (GAM-03; Fig. S2b), brown (GAM-04; Fig. S2b), and light yellowish-brown (GAM-05).
- (iii) Taung locality where 3 samples (TAU-01 to TAU-03) were collected from riverbeds (TAU-01 and TAU-03) and a hill (TAU-02), showing similar fine-grained texture with colors varying from light olive-brown (TAU-01; Fig. S3a), red (TAU-02; Fig. S3b), and olive-grey (TAU-03; Fig. S3c).

### Analytical methods

Major and trace elements as well as pH and OM analyses were conducted at the University of Johannesburg, Spectrum laboratory, whereas anions were determined by ion chromatography (IC) at the Agricultural Research Council, Soil, Climate and Water (ARC—SCW) laboratory in Pretoria, South Africa.

(i) Major elements analysis procedure: 2 g of each sample was kept overnight in a drying oven at 105 ˚C and then heated for 30 min at 930 ˚C. Glass discs were prepared by fusing the ignited samples weighing ~ 0.7 g together with 0.1 g of lithium nitrate (LiNO_3_) and 6 g of 50/50 flux consisting of 49.8% lithium tetraborate (Li_2_B_4_O_7_), 49.8% lithium metaborate (LiBO_2_) and 0.5% lithium bromide (LiBr) at 1050 ˚C. Major oxide elements determined were Al_2_O_3_, CaO, Fe_2_O_3_, K_2_O, MgO, Na_2_O, and SiO_2_ using X-Ray Fluorescence (XRF) analytical technique.
(ii) Trace elements analysis procedure: 0.30 g of each sample was dissolved in 9.0 ml of nitric acid (HNO_3_) and 3.00 mL of hydrochloric acid (HCl) into a microwave digestion vessel liner and placed into Mars6 microwave for 60—120 min. Digested samples were cooled at room temperature, filtered, centrifuged, and stored in a volumetric flask. About 1.00 ml portion was pipetted and diluted to 10 ml standards and then taken for Inductively Coupled Plasma-Mass Spectrometry (ICP-MS) for trace elements characterization (As, Cr, Co, Cu, Pb, Mn, Ni, and Zn) (USEPA, 1995).
(iii) pH and OM content: for each sample, pH was determined in both ratios (i.e., sample:H_2_O ratio (1:2.5) and sample:KCl ratio (1:2.5)) (van Reeuwijk, 2002), and organic matter (OM) content was determined by measuring the weight loss before and after ashing at 430 °C (Rowell, 2000).
(iv) Bioaccessibility tests: Physiologically based extraction test (PBET) was used to determine the bioaccessible fraction (BAF) of some elements of concerns (As, Cr, Co, Cu, Mn, and Ni) in the studied samples (Hong et al., 2016; Momoh et al., 2013). PBET entails BAF extraction using a two-step extraction method, representing the stomach and intestinal phases, simulating the chemical conditions of human gastrointestinal tract. Percentage of oral bioaccessible fraction was calculated using$, \%BAF=\frac{{C}_{b}}{{C}_{t}} \times 100$, where, C_b_ is the concentration of trace element released from the sample using PBET assay via the stomach or intestinal phases; C_t_ is the pseudototal concentration of trace element in the consumed material.

### Data processing and statistical analysis

#### Geochemical data and comparison to recommended standard values

Concentrations of major (mg/kg) and trace elements (mg/kg) as well as anions (mg/kg) were further compared with their recommended daily allowance (RDA), adequate intake (AI), and tolerable upper intake level (UL) standards for adults and pregnant women set values by the World Health Organisation (WHO) and Food and Agriculture Organisation (FAO) as presented in Table 1, to infer the aptness of the geophagic materials by ingestion.

**Table 1:** Recommended daily intake standards (in mg/kg) for adults and pregnant women

Recommended standards	Variables	Adults	Pregnancy	References
Recommended daily allowance (RDA)	Aluminum (Al)	0.10 – 0.12	–	ATSDR (2011)
	Calcium (Ca)	1 200 – 1 300	1 000 – 1 300	IOM (1997)
	Magnesium (Mg)	240 – 420	350 – 400	
	Copper (Cu)	0.9	1	IOM (2001)
	Iron (Fe)	8 – 18	27	
	Silica (Si)	12	19	
	Zinc (Zn)	8 – 11	11	
Adequate intake (AI)	Chloride (Cl^−^)	100	100	IOM (2005)
	Nitrate (NO_3-_)	3.7	3.7	
	Nitrite (NO_2-_)	0.06	0.06	
	Potassium (K)	2 300 – 3 400	2 600 – 2 900	
	Sodium (Na)	1 200 – 1 500	1500	
	Chromium (Cr)	0.025 – 0.035	0.029 – 0.030	IOM (2001)
	Manganese (Mn)	1.8 – 2.3	2	
	Cobalt (Co)	0.003 – 0.008	0.003 – 0.008	ATSDR (2004)
	Sulphate (SO_4_^2−^)	14	14	NRC (2005)
Tolerable upper intake level (UL)	Arsenic (As)	0.0005 – 0.00081	0.0005 – 0.00081	IOM (2001)
	Nickel (Ni)	1	1	
	Lead (Pb)	0.01	0.01	ATSDR (2017)

**Non-carcinogenic risk assessment** was assessed using parameters such as estimated daily intake (EDI), hazard quotient (HQ), and hazard index (HI) proposed by USEPA (1989).

**Estimated daily intake** was used to assess the average daily elements loading into the human system of specified body weight of the geophagic individuals (Meseret et al., 2020). The EDI values were calculated by ${EDI}_{ingestion}= \frac{C\times IR}{BW}$, where, C was the average weighted of element concentrations in geophagic materials (mg/kg), IR (ingestion rate) the average daily consumption of material (g/day person), and BW the average body weight (kg). Based on a geophagy survey conducted in the FTLM area, the average daily ingestion for adults was 170 g/day with an average body weight of 70 kg (Malepe, 2022).

**Hazard Quotient** depends on EDI and oral reference dose (RfD) to assess non-carcinogenic risk to humans from a long-term exposure of elements from the consumed materials, being calculated by $HQ= \frac{EDI}{RfD}$, where, EDI and RfD are expressed as mg/kg/day. RfD estimates daily exposure to which a person is expected without any significant risk of harmful effects during a lifetime (Meseret et al., 2020). The safety limit for HQ < 1 indicates that no potential health risks are expected from exposure, whereas HQ > 1 indicates potential non-carcinogenic effects (Candeias et al., 2020).

**Hazard Index** is the sum of HQ values of all elements and determined using $HI=\sum_{i=1}^{n}HQi$*,* given that i is the ingestion route for all elements of concern in the geophagic materials. HQ values evaluate the overall non-carcinogenic health risk through more than one element. If HI value of > 1, a high possibility of exposed individuals to experience adverse health effects (Kortei et al., 2020).

## Results

### Geochemical composition and physicochemical nature of the samples

Major elements composition is presented in Table 2. Results showed that all samples were characterized by a significant (*p* < 0.05) compositional variation, with SiO_2_ (21.8% to 67.9%), Al_2_O_3_ (1.2% to 24.9%), Fe_2_O_3_ (2.5% to 26.4%), and MgO (0.6% to 31.7%), and moderate to minor variation of CaO (0.2% to 6.7%), K_2_O (0.3% to 10.7%) and Na_2_O (0.4% to 2.7%). Average composition of various major elements (in mg/kg) were ranked as follows: Si (247,760) > Al (75,252) > Fe (66,927) > Mg (40,333) > Ca (17,343) > K (14,822) > Na (7774). A significant (*p* < 0.05) variation in the composition has also been noticed with the following trace elements (Table 2): Mn (33.52 mg/kg to 533 mg/kg), Cr (25.0 mg/kg to 357 mg/kg), Ni (17.55 mg/kg to 77.7 mg/kg), Zn (2.50 mg/kg to 65.6 mg/kg) and Cu (2.05 mg/kg to 38.52 mg/kg) and a minor variation in concentrations of Co (3.89 mg/kg to 16.05 mg/kg), Pb (0.18 mg/kg to 2.68 mg/kg) and As (0.32 mg/kg to 4.37 mg/kg). Average composition of trace elements (in mg/kg) were ranked Mn (257) > Cr (105) > Ni (40.5) > Zn (21.8) > Cu (14.4) > Co (9.85) > Pb (1.85) > As (1.56).

**Table 2:** Major and trace elements in studied geophagic samples (in mg/kg)

	DRI-01	DRI-02	DRI-03	DRI-04	GAM-01	GAM-02	GAM-03	GAM-04	GAM-05	TAU-01	TAU-02	TAU-03	Average
Al	6 456	59 694	88 271	89 858	75 729	60 593	80 597	80 650	69 854	118 488	41 172	131 665	75252
Ca	47 956	20 512	14 223	13 008	4 646	15 795	22 084	20 083	40 095	2 787	5 646	1 286	17343
Fe	17 555	76 025	56 651	61 407	41 195	47 979	67 702	76 235	58 470	54 343	184 921	60 638	66927
K	ND	2 075	5 977	7 720	16 603	12 784	8 966	5 728	8 883	30 965	29 304	34 036	14822
Mg	190 913	75 098	26 480	26 842	19 966	10 194	16 347	64 361	24 369	6 997	18 639	3 800	40333
Na	ND	2 893	6 899	7 493	3 561	4 600	4 525	16 840	20,030	5 490	ND	5 416	7774
Si	101 681	240 389	266 008	256 424	287 139	317 199	260 070	232 020	254 367	264 465	239 454	253 899	247760
As	0.32	0.33	0.57	0.78	1.55	1.57	1.74	0.36	1.84	4.37	2.52	2.81	1.56
Cr	135	357	168	146	25.9	34.87	98.72	146	35.85	25.02	56.18	26.52	105
Co	5.48	15.52	16.02	14.61	8.58	6.91	16.05	11.33	11.72	3.89	4.08	4.01	9.85
Cu	2.05	11.98	10.87	11.54	14.87	16.35	26.01	38.52	18.06	11.67	2.21	8.51	14.4
Pb	0.18	0.59	2.02	2.55	2.68	2.62	2.64	0.95	2.65	2.15	1.1	2.02	1.85
Mn	100	345	333	351	441	265	363	143	533	33.52	119	58.05	257
Ni	42.6	77.7	54.74	52.25	70.89	18.94	42.11	35.53	20.71	17.55	22.73	30.65	40.5
Zn	8	13.32	13.93	14.07	65.6	65.08	7.53	19.92	14.9	16.28	2.5	21.03	21.8

*ND*-not detected

Anions composition, and pH and OM content of the studied samples are presented in Table 3. Except for concentrations of NO_2_^−^ (0.01 mg/kg to 0.38 mg/kg) which showed minor variations (p > 0.05), concentrations of Cl^−^ (1.31 mg/kg to 1438 mg/kg), SO_4_^2−^ (1.76 mg/kg to 100 mg/kg) and NO_3_^−^ (1.74 mg/kg to 38.5 mg/kg) revealed significant variations (p < 0.05). Average concentrations of anions were ranked as follows: Cl^−^ > SO_4_^2−^ > NO_3_^−^ > NO_2_^−^. The pH_KCl_ values were lower than pH_H2O_, with a minimum of 5.04 in sample TAU-01 and a maximum of 8.03 in sample DRI-01. Minimum pH_H2O_ value of 6.8 was found in sample DRI-03 and a maximum pH_H2O_ value of 9.22 in sample GAM-05. Organic matter content ranged from 0.21% in sample TAU-01 to 1.7% in sample DRI-01.

**Table 3:** Anions (in mg/kg), pH and organic matter (in %) content of the studied geophagic materials

	Cl^−^	NO_3-_	NO_2-_	SO_4_^2−^	pH (H_2_0)	pH (KCl)	OM
DRI-01	1.31	1.74	0.05	3.58	8.68	8.03	1.7
DRI-02	5.26	6.78	0.02	15.4	8.33	7.3	1.46
DRI-03	637	38.5	0	22.3	6.8	6.2	0.73
DRI-04	1438	31.8	0	100	7.12	6.64	0.53
GAM-01	57.5	2.64	0	24.4	8.33	6.57	0.54
GAM-02	4.49	5.64	0.38	9.24	8.47	7.72	0.53
GAM-03	4.02	8.14	0.14	7.54	8.16	6.96	0.83
GAM-04	5.88	9.92	0.003	12.73	8.54	6.94	0.31
GAM-05	2.63	4.58	0.01	3.65	9.22	7.18	0.25
TAU-01	1.48	2.14	0.01	2.29	7.06	5.04	0.41
TAU-02	2.92	1.74	0.04	2.24	8.37	6.1	0.21
TAU-03	1.36	3.31	0.012	1.76	7.67	6.29	0.34
Average	180	7.74	0.056	17.1	8.06	6.75	0.65

**Fig. 2:**
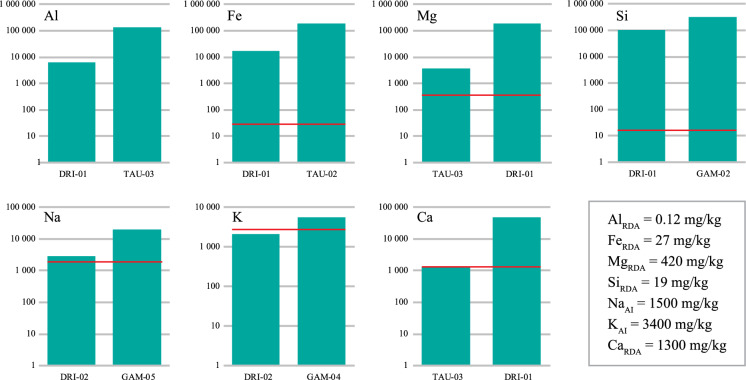
Major elements composition showing minimum (left) and maximum (right) concentrations of Al, Fe, Mg, Si, Na, K, and Ca (logarithmic scale). Red line represents AI and RDA standards limits described in the box

Major elements showed that all samples (*n* = 12) presented Al, Fe, Mg, and Si concentrations above their recommended standard allowance (RDA) values for adults and pregnant women (IOM, 1997, 2001; ATSDR, 2011) (Fig. 2a–d). Concentrations of Na were higher than adequate intake (AI) standard (Fig. 2e), except for samples DRI-01 and TAU-01. Similar results were observed for K concentrations, except for samples DRI-01 and DRI-02 (Fig. 2f). In the case of Ca, except for sample TAU-03, which was characterized by concentrations (1286 mg/kg) within the RDA standard, its concentration in other samples (*n* = 11) were all above the RDA value (Fig. 2g).

**Fig. 3:**
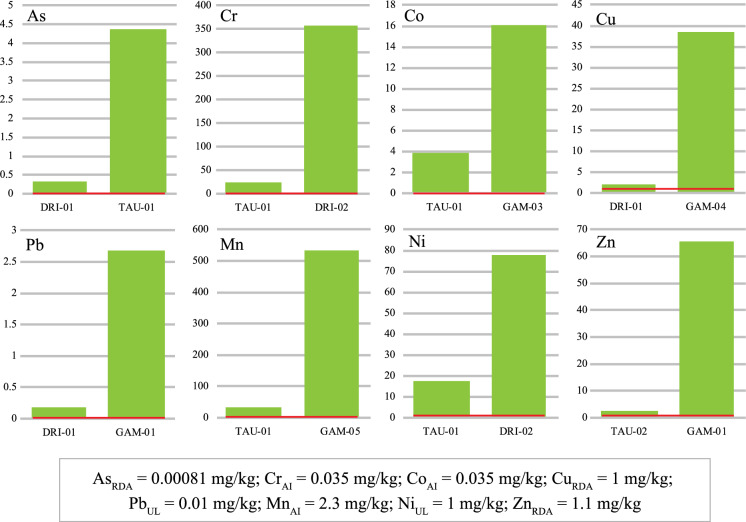
Trace elements compositions showing minimum (left) and maximum (right) concentrations of As, Cr, Co, Cu, Pb, Mn, Ni and Zn concentrations (in mg/kg). Red line represents guideline limits

Trace elements such as Cu, Ni, and Zn were selected because of their biological significance, whereas As, Cr, Co, Pb, and Mn were selected due to their potentially toxic nature (Fig. 3a–h). When compared with their correspondent AI, RDA, and upper intake level (UL) standards, proposed by IOM (2001) and ATSDR (2017) for adults and pregnant women, the minimum and maximum concentrations results showed that concentrations of As, Cr, Co, Cu, Pb, Mn, and Ni were significantly high in all samples (*n* = 12) (Fig. 3a–g). However, in the case of Zn, two samples (TAU-02 and GAM-03) showed lower concentrations (2.50 mg/kg and 7.53 mg/kg, respectively) than the RDA values, meanwhile sample DRI-01 was within the RDA range (Fig. 3h).

**Fig. 4:**
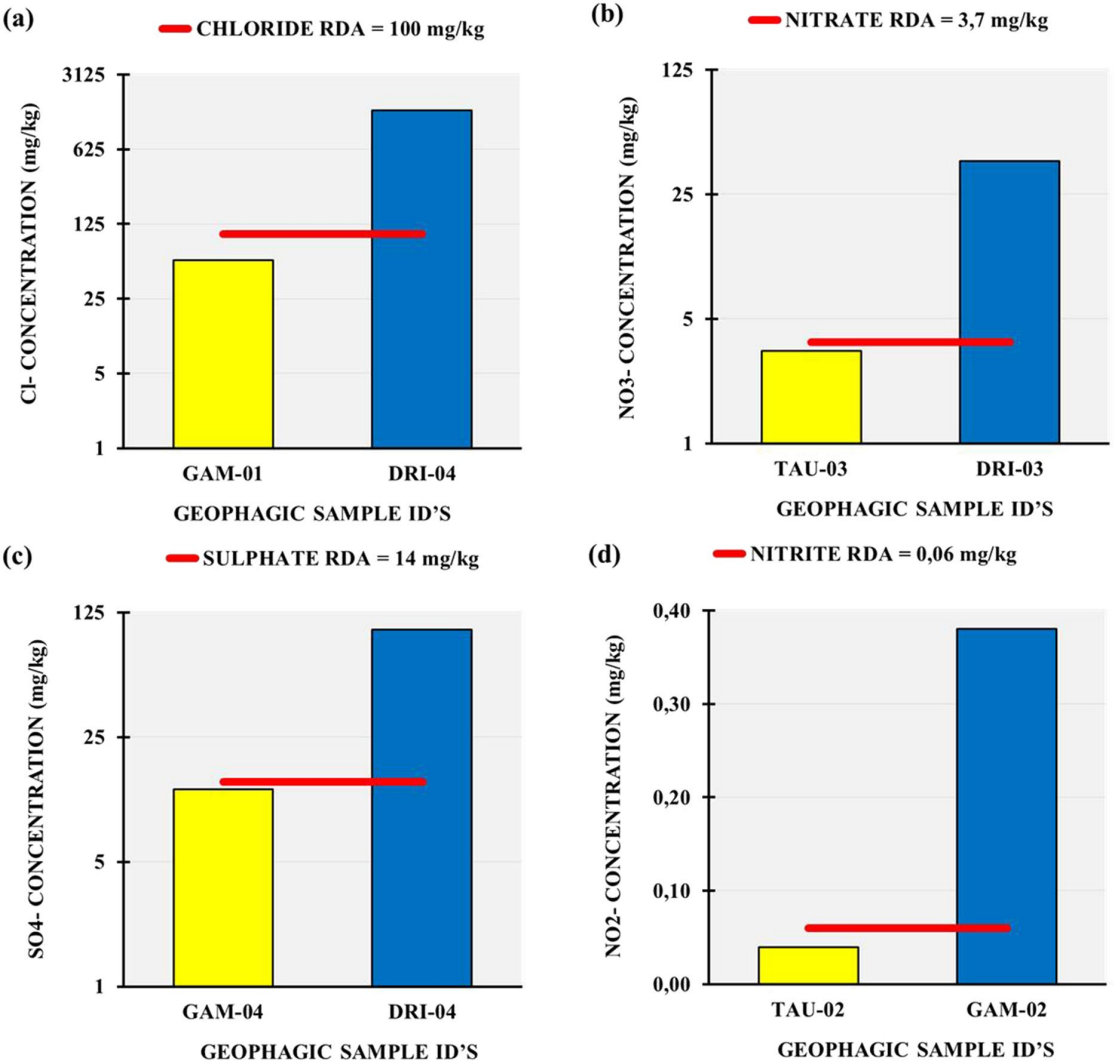
Anions compositions showing minimum (below RDA) (left) and maximum (right) (above RDA) Cl^−^, NO_3_^−^, SO_4_^2−^ and NO_2_^−^ concentrations (in mg/kg). Red line represents guideline limits (RDA)

Results of the anions analysis (Table 3) showed that 2 samples (DRI-03 and DRI-04) were characterized by high Cl^−^ concentrations (up to 1438 mg/kg in DRI-04) above their RDA standard, while the rest of samples showed relatively lower values ranging between 1.31 and 57.5 mg/kg (Fig. 4a). The same samples (DRI-03 and DRI-04) as well as DRI-02 and GAM-02 to 05 showed higher concentrations of NO_3_^−^ than RDA standard, with the highest value (up to 38.5 mg/kg) observed in sample DRI-03 (Fig. 4b). Higher concentrations of SO_4_^2−^ than RDA were also observed in the same samples DRI-02, DRI-03, DRI-04 as above in addition to sample GAM-01 (Fig. 4c). In the case of NO_2_^−^, however higher concentrations (0.383 mg/kg and 0.148 mg/kg) than the RDA value were observed only in samples GAM-02 and GAM-03 respectively (Fig. 4d).

### Health risk assessment

Estimated daily intake (EDI)of the consumed geophagic materials as well as values of reference dose (RfD) are presented in Table 4. All samples revealed higher EDI values of As (> 0.001 mg/kg/day), Cr (> 0.063 mg/kg/day), Mn (> 0.081 mg/kg/day) and Ni (> 0.043 mg/kg/day) than their RfD values. All samples revealed lower EDI values with the RfD value of Zn up to 0.159 mg/kg/day.

**Table 4:** Estimated daily intake (EDI), reference dose (RfD) (in mg/kg/day) and hazard index (HI) content for adults through ingesting the studied geophagic materials

	As	Cr	Co	Cu	Pb	Mn	Ni	Zn	HI
DRI-01	0.001	0.328	0.013	0.005	0.00004	0.234	0.103	0.019	129
DRI-02	0.001	0.867	0.038	0.029	0.001	0.838	0.189	0.033	342
DRI-03	0.001	0.408	0.039	0.026	0.005	0.809	0.133	0.034	188
DRI-04	0.002	0.355	0.035	0.028	0.006	0.852	0.127	0.034	173
GAM-01	0.004	0.063	0.021	0.036	0.006	1.071	0.172	0.159	93
GAM-02	0.004	0.085	0.017	0.04	0.006	0.644	0.046	0.039	76
GAM-03	0.004	0.24	0.030	0.063	0.006	0.882	0.102	0.048	145
GAM-04	0.001	0.354	0.028	0.094	0.002	0.347	0.086	0.032	148
GAM-05	0.004	0.087	0.028	0.044	0.006	1.294	0.050	0.036	107
TAU-01	0.011	0.061	0.009	0.028	0.005	0.081	0.043	0.031	65
TAU-02	0.001	0.064	0.01	0.005	0.003	0.289	0.055	0.006	83
TAU-03	0.007	0.064	0.01	0.021	0.005	0.141	0.074	0.051	57
RfD	0.0003	0.003	0.01	0.02	0.004	0.024	0.02	0.3	

**Fig. 5:**
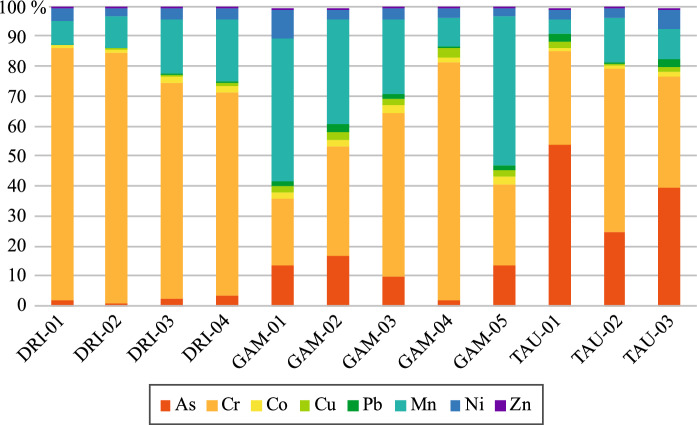
Estimated Hazard Quotient (HQ) in the studied samples

Estimated Hazard Quotient (HQ) values for As, Cr, Co, Cu, Pb, Mn, Ni, and Zn, are presented Fig. 5. Results showed that HQ ingestion values for Cr and Mn were significantly (p < 0.05) higher than 1 in all samples, followed by As and Ni, with maximum values of 289, 53, 35 and 9.4, respectively. All three samples from the Taung locality (TAU-01 to TAU-03) showed HQ values < 1 for Co (up to 0.99) and Zn (up to 0.17).

Hazard Index (HI) values for adults by geophagic materials ingestion results were significantly higher than safety limit (HI > 1) in all samples (*n* = 12), with minimum of 129, 76, and 57, in samples DRI-01, GAM-02 and TAU-03, respectively (Table 4).

### Bioaccessibility test

**Fig. 6:**
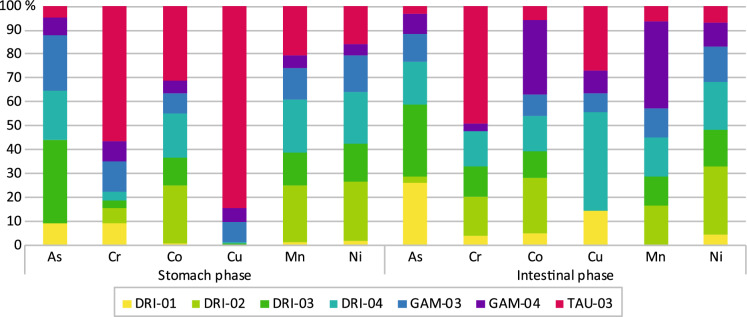
Bioaccessible fractions (%BAF) of As, Cr, Co, Cu, Mn, and Ni for stomach and intestinal phases

Samples DRI-01 to DRI-04, GAM-03 to GAM-04 and TAU-03, were selected for the bioaccessibility test (Fig. 6), due to high concentration of As, Cr, Co, Cu, Mn, and Ni, relative to their recommended daily standards intake (IOM, 2001; ATSDR, 2004). Analyzed trace elements (Fig. 6), revealed that the % bioaccessible fraction (%BAF) for Mn was the highest in both stomach and intestinal phases, ranging from 3.26 to 62.7% and 10.9 to 62.6%, respectively. Other elements revealed, %BAF for Cu (0.02 to 14.2% in the stomach; 0.09 to 9.4% in intestinal), Ni (1.22 to 15.3% in the stomach; 2.09 to 12.6% in intestinal) and As (0.005 to 11.9% in the stomach; 1.2 to 12.9% in intestinal). Cu %BAF was generally very low, except for sample TAU-03 with 14.0%. Sample TAU-03 showed the highest Cr %BAF for stomach and intestinal phases with 12.9 and 9.6%, respectively.

## Discussion

Geophagic materials are considered as a primary nutritional source of essential elements by geophagic individuals. The physiological and/or nutritional aspects are amongst the common motivations that geophagic individuals use to justify the practice. However, geophagic materials might contain potentially toxic elements (Skalnaya & Skalny, 2018), which can be detrimental to health, depending on their concentrations and distribution (Selinus et al., 2005). Essential elements concentrations must be within the recommended daily intake standards that potentially aid human health, while excess or deficiency of such elements might induce serious health outcomes (Steffan et al., 2018).

Aluminum is known to be a toxic element, and exposure to high concentrations of this element through different pathways, including ingestion, can promote health issues, such as Alzheimer’s disease, as reported by several studies (e.g., Alasfar & Isaifan, 2021). In the case of the present study, all samples showed Al concentrations significantly (*p* > 0.05) higher (6456 mg/kg to 131,665 mg/kg) than the proposed RDA (Fig. 2a; Tables 1–2), suggesting that ingestion of such materials might represent a potential health risk to the consumers. The need for Fe supplementation is one of the known motivations for geophagy, especially by pregnant women (Kambunga et al., 2019a), due to an increased demand for blood volume necessary to cover blood loss during childbirth (van Onselen et al., 2015). Iron is essential for complex processes necessary for a healthy life, e.g., transfer oxygen to various tissues and production of red blood cells and hormones (van Onselen et al., 2015). However, excessive intake of Fe, might lead to serious health issues such as liver damage in pregnant women (Okereafor et al., 2016), decreased growth, increased inflammatory markers and diarrhea (Lönnerdal, 2017). In addition, the elements might interact with other trace elements such as Cu and impact on their absorption, although this may depend on pH, bioacessibility, consumption frequency, and amount of the consumed materials (George & Abiodun, 2012; Lee et al., 2021)). Therefore, consumption of the studied geophagic materials, with concentrations of Fe (17,555 mg/kg to 184,921 mg/kg) exceeding the proposed RDA (Fig. 2b; Table 1) in all samples, might be harmful to the health of the consumers.

Magnesium is the second most abundant intracellular cation after K, being essential for bone mineralization, muscular relaxation, and several other cellular functions (Al Alawi et al., 2018; Fiorentini et al., 2021). However, Mg intake should be within the RDA of 240 to 420 mg/kg (Table 1). In the case of the studied samples, Mg concentration varied from 3800 mg/kg to 190,913 mg/kg, above the proposed RDA (Fig. 2c; Table 1). Excess of Mg intake could result in toxic effects characterized by low blood pressure and sugar levels (Soetan et al., 2010), suggesting that consumption of the studied samples might be harmful to the consumers.

In the case of Si, the amorphous forms seem to be more soluble than the crystalline ones, therefore less toxic (Brunner et al., 2006). The health issues related to amorphous forms of Si are still not well understood (Pavan et al., 2019). However, the ingestion of the crystalline forms of Si and/or inhalation of nanoparticles during the collection of the material in the field can cause several health issues including lung cancer, neurotoxicity, fibrosis and renal injury amongst many others (Vareda et al., 2021). Silica in the studied samples was the dominant major chemical element (Table 2), with concentrations significantly (*p* < 0.005) higher (101,681 mg/kg to 316,409 mg/kg) than the proposed RDA (Fig. 2d; Table 1). Therefore, consumption of such materials might induce potential health issues. Although Na might provide essential benefits to the human body, its excess intake can lead to serious health issues including high blood pressure (Cook et al., 2020). Concentration of Na in most studied samples (*n* = 10) were above the proposed RDA (Fig. 2e; Table 1), hence might suggest a potential health hazard to consumers.

Amongst the essential functions of potassium is to regulate blood pressure and heartbeat, maintains fluid balance and helps muscles contraction (Gomes & Silva, 2007). Studied samples revealed high concentrations of K (up to 34,036 mg/kg) when compared to the RDA (Fig. 2f; Table 1). Apart from serious impact on blood pressure leading to cardiovascular diseases (Weaver, 2013), such high concentrations of K, may cause toxicity, which has been associated with a serious rare condition known as Hyperkalemia (George & Ndip, 2011) depending on the frequency and amount of material consumed. Calcium is an essential element for the formation of bones and teeth structures (Gomes & Silva, 2007). This element is vital during pregnancy, being responsible for bone and skeletal development of the fetus (Wiley & Katz, 1998). Its concentration in studied samples (up to 47,956 mg/kg) was higher than the proposed RDA (Fig. 2g; Table 1) in most samples. Therefore, excessive, and frequent intake of such material with high amount of calcium might lead to serious health issues including hypercalcemia (Machado et al., 2015).

In the case of trace elements, As is classified as carcinogenic type 1, with no known biological functions (). Its adverse health effects are influenced by its dominant oxidation inorganic forms (arsenate (As^5^) and arsenite (As^3^)) (IOM, 2001). Its concentration (0.32 mg/kg and 4.37 mg/kg) significantly higher than the proposed UL level (Fig. 3a; Table 1), might represent a serious health risk to the consumers of the materials. Acute exposure to As is characterized by headaches, abdominal pains, vomiting, diarrhea, muscular pains, and numbness (Kamunda et al., 2016), whereas chronic exposure through ingestion can cause miscarriages and premature childbirth in pregnant women (). Chromium is also classified as carcinogenic and mutagenic to humans with no recognized biological functions (). Concentrations of Cr (25 mg/kg to 357 mg/kg) in all the studied samples were higher than the proposed AI level (Fig. 3b; Table 1) and could be associated with detrimental health effects on geophagic individuals. Excess Cr intake is associated with severe irritation of the eye, skin, digestive, and respiratory tract with possible caustic burns when consumed (Shekhawat et al., 2015). Other signs of Cr toxicity include hypertension, back pains, malformations, skeletal defects, and mortality during pregnancy (Han et al., 2017), depending on its inorganic oxidation form (3 + and 6 +), and solubility of Cr compounds (Shekhawat et al., 2015). Cobalt is known for its biological importance including its role as metal constituent of vitamin B12, however excessive intake might result in potential health hazards related to cardiovascular, neurological, and endocrine systems (Leyssens et al., 2017). The concentrations (3.89 mg/kg to 11.7 mg/kg) of cobalt in all the studied samples were significantly (*p* < 0.05) above the proposed AI level (Fig. 3c; Table 1), hence might be considered a health threat the consumers. Although Cu is considered essential for humans to function at a tolerable level, this element becomes toxic when in excess (WHO, 1996). Therefore, the high Cu concentration (2.05 to 38.5 mg/kg) above the proposed RDA (Fig. 3d; Table 1) revealed in the studied samples, might lead to short and/or long-term health issues such as fatigue, loss of concentration, liver damage and learning disabilities (Soetan et al., 2010. Lead is classified as carcinogen () with a range of symptoms and toxic effects including anemia, impacts on nervous system, premature birth, babies with a low birth weight and even death (Wani et al., 2015). Studied samples Pb concentration (0.18 mg/kg to 2.68 mg/kg) were above the proposed UL level (Fig. 3e; Table 1), might represent a serious health threat the consumers of the material. Manganese is necessary for normal functions such as bones mineralisation, protein and energy metabolism, cellular protection from damaging free radical species amongst others (e.g. ATSDR, 2012). However, its excessive intake might result in the development of the so-called “Manganism,” which is a neurological condition characterized by some symptoms like Parkinson’s disease (Harischandra et al., 2019), although the absorption of Mn is influenced by gender, age, and bioavailability of other elements such as Ca, Fe, and P (IOM, 2001). Mn concentration (33.5 mg/kg to 533 mg/kg) in the studied samples were significantly higher than the proposed AI level (Fig. 3f; Table 1) suggesting potential health impact on the consumers. The high concentrations (17.6 mg/kg to 77.8 mg/kg) of Ni in studied samples was above the proposed UL level (Fig. 3g; Table 1), what might represent a hazard to the geophagic individual’s health. Excess intake of Ni has been related to adverse health risks such as nausea, vomiting, diarrhea, headache, increased red blood cells, shortness of breath, and heart failure leading to death (Kumar & Trivedi, 2016). Although Zn is essential for the function of immune system (Maywald & Rink, 2022), excess of this element was observed, in some of the studied samples (up to 65.6 mg/kg), above the proposed RDA (Fig. 3h; Table 1), which might result in potential health issues such as those related to the gastrointestinal (GI) system (Skalny et al., 2021). Furthermore, excess intake of Zn can disturb the availability of other elements in the body such as Cu, which can lead to its deficiency and related health issues (Brzóska et al., 2021).

Cl^−^ is the most dominant anion (average 180 mg/kg) in the studied samples (Table 3) and presented concentration (637 mg/kg and 1438 mg/kg) significantly (*p* < 0.05) higher than the proposed RDA value (100 mg/kg) in samples DRI-03 and DRI-04, respectively (Fig. 4a; Table 1). Frequent consumption of materials with such high Cl^−^ concentration might lead to the so-called hyperchloraemia, amongst other diseases (Turck et al., 2019).

The presence of nitrates (up to 38.5 mg/kg), nitrites (up to 0.38 mg/kg) and sulphate (up to 100 mg/kg) in most of the studied samples was above the proposed RDA standards (Fig. 4b-d; Table 1), which might result in toxic effects. According to Karwowska and Kononiuk (2020), nitrates and nitrites are not carcinogenic, however they might form carcinogens by reacting with other elements and, their high dietary intake might lead to toxicity in the form of methemoglobinemia.

The pH_KCl_ values in all consumed materials were lower than pH_H2O_ (Table 3), suggesting that these samples were positively charged (i.e., ΔpH (pH_H2O_)—pH_KCl_). The gap between pH in H_2_O (active or real acidity) and KCl (potential acidity) values makes it possible to determine the reserve (or total) acidity of the geophagic materials. The positive ΔpH values (reserved acidity) indicated that the exchange complex of the samples was dominated by positive charges (Tan, 1982). Therefore, samples that contained a considerable amount of reserved acidity were favorable to chemical reactions in the stomach. As a result of the stomach pH = 2, a possible reaction could occur (Oomen et al., 2000). These may depend on the stomach residence time (~ 2 h) of the ingested material, which is insufficient for any significant reactions to occur. Lower pH in the intestines could result in the release of cations that may have been adsorbed on the exchange sites of the consumed material. The pH of these samples is unlikely to drop on the stomach pH because of the buffering capacity when consumed. The solubility of Fe and other cations in the GI tract increases with a decrease in pH (Okereafor et al., 2016; Young et al., 2008). Thus, consuming these samples may prevent the stomach pH from falling to levels that are favorable for Fe dissolution, thereby reducing its bioavailability to the consumers even when Fe concentration in the consumed materials was high. Studied samples pH_H2O_ values ranged from moderate (6.80) to very strong alkaline (9.22), higher than the gastric acid, which could be beneficial for heartburn soothing. In addition, these samples might not have a noticeable impact on essential elements and nutrients released in the GI tract, unless the consumed material is of a clay size to cause a chemical reaction (Kambunga et al., 2019a; Oomen et al., 2000).

The organic matter (OM) in some of the studied samples (DRI-01 to DRI-03 and GAM-03) was high (> 0.7%) (Table 3), suggesting that these samples are likely to harbor pathogenic bacteria, which might induce detrimental health outcomes. However, according to some authors (Ekosse et al., 2010; Msibi, 2014) the use of heat treatment of geophagic materials before consumption might help destroy harmful bacteria and pathogens present, hence reduce the risks.

### Health risk assessment

Estimated daily intake of As, Cr, Mn and Ni were relatively above their respective reference doses, in most samples (Table 4). Studied geophagic samples (*n* = 12) were considered not recommended for consumption, with HQ values > 1 for As (maximum = 35), Cr (maximum = 289), Mn (maximum = 53) and Ni (maximum = 9.4) (Fig. 5). These samples could induce non-carcinogenic health effects on consumers in the long term. Other elements, such as Co, Cu, Pb, and Zn had HQ values < 1 in some samples, did not show any significant health risk to adults by ingestion, their bioaccumulation after prolonged exposure may pose health risks. All the studied samples presented HI values significantly > 1 (Table 4), suggesting that these samples might pose potential health risk to geophagic individuals. Non-carcinogenic health risk indicated a potential health threat depending on factors such as dosage and chemical species as well as age, gender, genetics, and nutritional status of the consumers.

Bioaccessibility represents the fraction of the element released from the consumed material matrix into the GI tract, becoming available for absorption (Candeias et al., 2021). Studied geophagic materials showed that studied As, Co, Cr, Cu, Mn and Ni content was more bioaccessible in the stomach than in intestinal conditions. Different trends of the bioaccessibility between the simulated stomach and intestinal conditions were controlled by factors such as stomach pH, particle size, and elemental mobility (Kutalek et al., 2010). Except for Co and Mn, it is worth noting that As and Cr, as well as Cu and Ni showed the lowest %BAF (up to 16.0%) in the stomach and intestinal conditions (Fig. 6). The low %BAF of Cu and Ni in studied samples DRI-01 to DRI-04, GAM-03, GAM-04, and TAU-03 could contribute to elemental nutrition and alleviation deficiencies in geophagic individuals. However, continuous consumption of these materials with the presence of such elements over a long period of time may increase non-carcinogenic outcomes on geophagic individuals. Despite low %BAF of As and Cr, these samples can potentially pose a carcinogenic risk to the consumers due to their toxicity effects regardless of their composition.

## Conclusions

Geophagic materials are consumed with the motivations that they contain essential elements with several health benefits. However, the geochemical composition of the studied materials relative to their proposed recommended daily intake standards showed evidence in contrast with this notion. Excessive amounts of most essential elements (e.g., Fe, K, Mg) and anions (Cl^−^, SO_4_^2−^, NO_3_^−^, NO_2_^−^) above the proposed recommended daily intake in the geophagic samples suggested that studied samples were not suitable for human consumption and might induced health outcomes. Non-essential elements (e.g., Al, As, Cr, Co, Pb) which have no biological significance to human health, were also above the proposed daily intake standards, that can result in acute and chronic health risks when consumed. Non-carcinogenic health risk showed that geophagic individuals might be exposed to potential toxic elements above the safety limit (HI > 1), being at risk of developing long-term non-carcinogenic diseases. Bioaccessible fraction of As, Cr, Cu and Ni, revealed low values (< 16.0%). Alkaline pH in the samples may be beneficial for soothing heartburn. However, these samples might have the potential to increase the gastric juice pH, thereby decreasing the bioavailability of some elements, especially Fe, causing anemia. In addition, high OM content in the studied samples may harbor pathogens causing bacterial infections to geophagic individuals.

Based on the geochemical analysis findings, the studied samples revealed the potential to cause detrimental health effects to geophagic individuals. Therefore, the geophagic materials consumption should be discouraged amongst the population in the study arera. Moreover, due to little information and no documented studies on the geochemical composition of geophagic materials and their potential health hazards in the study area (FTLM), educational program awareness is recommended for geophagic individuals to understand the impact of this practice on their health and that of their children especially in the case of pregnant women.

## Supplementary Information

Below is the link to the electronic supplementary material.

Supplementary file1 (PDF 4520 KB)

## Data Availability

The authors confirm that the data generated in this study are presented in the manuscript. However, additional data generated, such as major oxides composition (in mass%) and is the concentration of trace element released from the sample using PBET assay via the stomach or intestinal phases (cb – bioaccessibility concentrations), can be provided upon request.
