# Supplementary material for: Geochemical composition and potential health risks of geophagic materials: an example from a rural area in the Limpopo Province of South Africa
Source: Environ Geochem Health. 2023 Jun 9;45(8):6305–22. doi: 10.1007/s10653-023-01551-6 (PMC10403411; doi:10.1007/s10653-023-01551-6)
Supplement: Supplementary file 1 — Supplementary file1 (PDF 4520 KB) [file 10653_2023_1551_MOESM1_ESM.pdf]

Geochemical composition and potential health risks of geophagic materials: an example from a rural area in the Limpopo Province of South Africa

Hassina Mouri[1]; Retshepile Evelyn Malepe[1]; Carla Candeias[2]

[1]Department of Geology, University of Johannesburg, South Africa
[2]GeoBioTec Research Unit, Geosciences Department, University of Aveiro, 3810-193 Aveiro, Portugal.

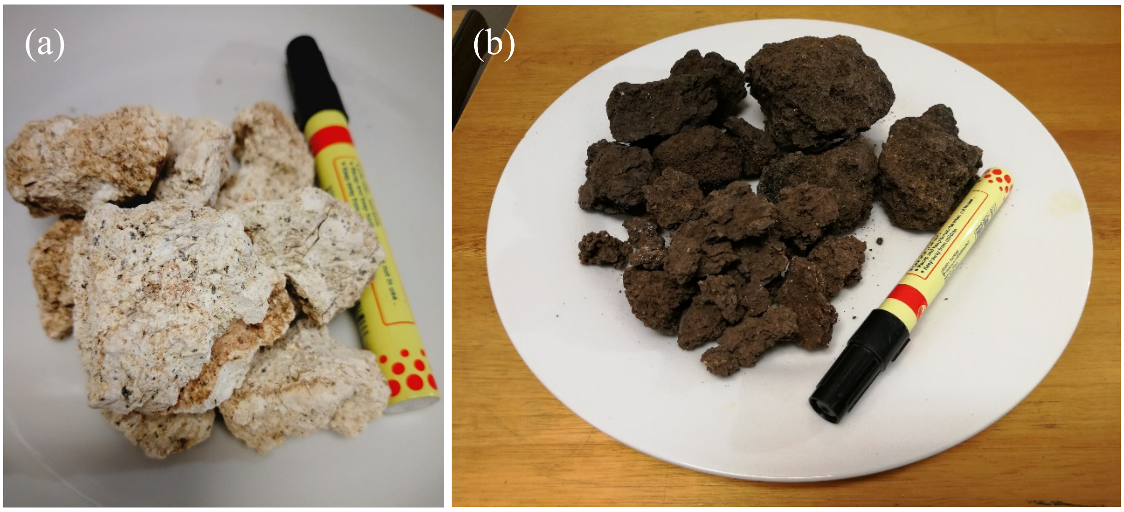

S1. Samples from Driekop locality, (a) DRI-01 and (b) DRI-02.

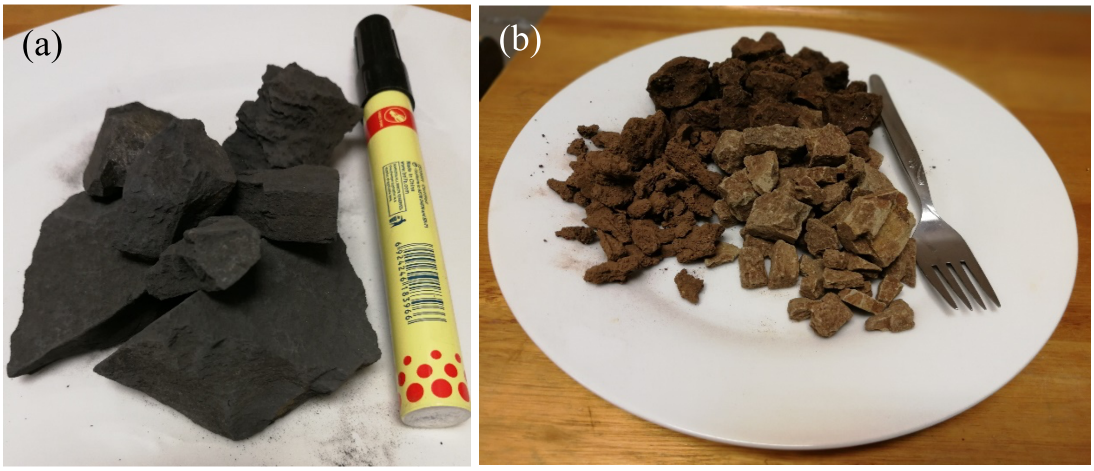

S2. Samples from the Ga-Motodi locality, (a) GAM-05, and (b) GAM-02 to GAM-04.

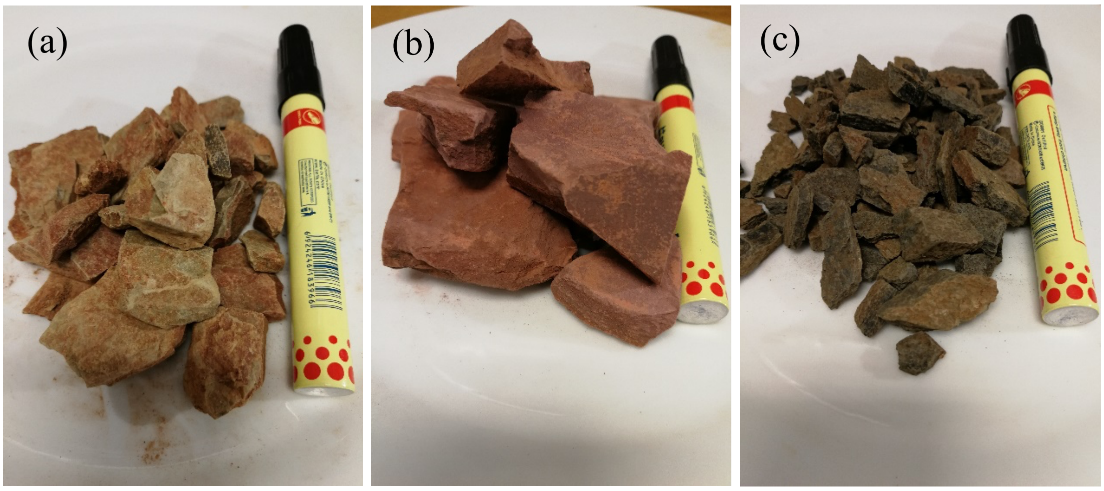

S3. Samples from the Taung locality, (a) TAU-01, (b) TAU-02, and (c) TAU-03.
